# Lessons Learned From Developing Dashboards to Support Decision-Making for Community Opioid Response by Community Stakeholders: Mixed Methods and Multisite Study

**DOI:** 10.2196/51525

**Published:** 2024-09-09

**Authors:** Naleef Fareed, Ramona G Olvera, Yiting Wang, Michael Hayes, Elizabeth Liz Larimore, Peter Balvanz, Ronald Langley, Corinna A Noel, Peter Rock, Daniel Redmond, Jessica Neufeld, Sarah Kosakowski, Daniel Harris, Marc LaRochelle, Timothy R Huerta, LaShawn Glasgow, Emmanuel Oga, Jennifer Villani, Elwin Wu

**Affiliations:** 1 Department of Biomedical Informatics College of Medicine The Ohio State University Columbus, OH United States; 2 Center for the Advancement of Team Science, Analytics, and Systems Thinking College of Medicine The Ohio State University Columbus, OH United States; 3 Department of Research Information Technology College of Medicine The Ohio State University Columbus, OH United States; 4 Research Triangle Institute Research Triangle Park, NC United States; 5 Center for Drug and Alcohol Research University of Kentucky Lexington, KY United States; 6 Clinical Addiction Research and Evaluation Unit Section of General Internal Medicine Boston Medical Center Boston, MA United States; 7 Department of Public and Ecosystem Health Cornell University Ithaca, NY United States; 8 Institute for Biomedical Informatics University of Kentucky Kentucky, KY United States; 9 Social Intervention Group School of Social Work Columbia University New York, NY United States; 10 Institute for Pharmaceutical Outcomes and Policy University of Kentucky Lexington, KY United States; 11 National Institute on Drug Abuse Bethesda, MD United States

**Keywords:** data visualizations, dashboards, public health, overdose epidemic, human-centered design

## Abstract

**Background:**

Data dashboards are published tools that present visualizations; they are increasingly used to display data about behavioral health, social determinants of health, and chronic and infectious disease risks to inform or support public health endeavors. Dashboards can be an evidence-based approach used by communities to influence decision-making in health care for specific populations. Despite widespread use, evidence on how to best design and use dashboards in the public health realm is limited. There is also a notable dearth of studies that examine and document the complexity and heterogeneity of dashboards in community settings.

**Objective:**

Community stakeholders engaged in the community response to the opioid overdose crisis could benefit from the use of data dashboards for decision-making. As part of the Communities That HEAL (CTH) intervention, community data dashboards were created for stakeholders to support decision-making. We assessed stakeholders’ perceptions of the usability and use of the CTH dashboards for decision-making.

**Methods:**

We conducted a mixed methods assessment between June and July 2021 on the use of CTH dashboards. We administered the System Usability Scale (SUS) and conducted semistructured group interviews with users in 33 communities across 4 states of the United States. The SUS comprises 10 five-point Likert-scale questions measuring usability, each scored from 0 to 4. The interview guides were informed by the technology adoption model (TAM) and focused on perceived usefulness, perceived ease of use, intention to use, and contextual factors.

**Results:**

Overall, 62 users of the CTH dashboards completed the SUS and interviews. SUS scores (grand mean 73, SD 4.6) indicated that CTH dashboards were within the acceptable range for usability. From the qualitative interview data, we inductively created subthemes within the 4 dimensions of the TAM to contextualize stakeholders’ perceptions of the dashboard’s usefulness and ease of use, their intention to use, and contextual factors. These data also highlighted gaps in knowledge, design, and use, which could help focus efforts to improve the use and comprehension of dashboards by stakeholders.

**Conclusions:**

We present a set of prioritized gaps identified by our national group and list a set of lessons learned for improved data dashboard design and use for community stakeholders. Findings from our novel application of both the SUS and TAM provide insights and highlight important gaps and lessons learned to inform the design of data dashboards for use by decision-making community stakeholders.

**Trial Registration:**

ClinicalTrials.gov NCT04111939; https://clinicaltrials.gov/study/NCT04111939

## Introduction

### Background

Data dashboards are tools, often published digitally on websites or dedicated apps, that present visualizations; they are increasingly used to display data about behavioral health, social determinants of health, chronic and infectious disease risks, and environmental risks to inform or support public health endeavors [1-4]. Dashboards can be an evidence-based approach used by communities to influence public awareness and decision-making and to focus the provision of resources and interventions in health care toward specific populations [2,3,5-8]. For example, local public health agencies across the nation have communicated health data about the COVID-19 pandemic through dashboards, using these as tools to generate awareness and motivate behavior change (eg, adherence to public health guidelines) [9].

Despite widespread use, evidence on how to best design and use dashboards in the public health realm is limited [1,9-12]. There is also a notable dearth of studies that examine and document the complexity and heterogeneity of dashboards in community settings. Experiences from the Healing Communities Study (HCS) provided an opportunity to empirically learn how dashboards and health data visualizations can be assessed to inform design and use, especially among community end users.

The HCS is implementing the Communities That HEAL (CTH) intervention aimed at reducing overdose deaths by working with community coalitions in selected counties and cities highly affected by opioid deaths in each state [13]. In January 2020, as part of the community engagement component of the CTH, 4 research sites followed a common protocol to develop community dashboards to support community coalitions in selecting evidence-based practices (EBPs) to reduce opioid overdose deaths in their respective communities. The protocol stipulated which key metrics to present to each community regarding opioid overdose deaths and associated factors that may contribute to their prevalence [14]. The CTH intervention protocol involved dashboard cocreation by HCS researchers and community stakeholders from each community that incorporated the principles of user-centered design [15].

We define community stakeholders for this analysis as individuals in the community who are engaged in fighting the overdose crisis, including coalition members (eg, county public health officials and behavioral health practitioners) and community research staff. The CTH intervention envisioned community-tailored dashboards as a tool that coalition members would use to discuss and understand baseline conditions and trends, to inform EBP selections, and monitor research outcomes of interest to the community. Community research staff, often with preexisting ties to the community, were to support and lead coalitions through the use of data dashboards for decision-making and ongoing monitoring. These stakeholders were the anticipated dashboard end users who were not expected to have expertise in the use of dashboards, even though community research staff had additional training in community engagement and overall study protocols. Our research team oversaw the design, implementation, maintenance, and evolution of the dashboards used by these stakeholders.

The CTH dashboard cocreation involved, at a minimum, iterative show-and-tell sessions in which feedback from community stakeholders was provided on wireframes with the goal of refining the dashboard and its ability to align with specific objectives (eg, to address local challenges in fighting the opioid crisis and to highlight key performance indicators). Although the CTH intervention required the same specific core components of the dashboards (eg, use of predetermined metrics, annotations for metrics, and granularity of the data presented), communities could incorporate unique components based on preferences and resources, such as displaying local data (eg, county opioid overdose death rates) acquired by the HCS. Thus, dashboards varied in layout, interface, and content across the 4 sites.

### Objectives

To elucidate lessons learned from providing dashboards to the coalitions and community stakeholders, we investigated the following questions on the CTH dashboards: Are the CTH dashboards usable and useful for community decision-making? Are the dashboards easy to use and understand? Will the dashboards be used in the long term, and, if yes, for what purposes will they be used? Our study is novel because of four specific areas: (1) the use of a qualitative approach (instead of a quantitative approach) to expound on the technology adoption model (TAM) constructs, informing existing perspectives on dashboard usability; (2) the investigation of dashboard usability across 67 diverse communities; (3) the generation of themes and subthemes on the usability of a dashboard in the substance abuse domain; and (4) the identification of common themes on dashboard usability from a TAM and System Usability Scale (SUS) perspective [16,17] by comparing feedback from 4 unique applications of a dashboard that was tailored to the needs of end users. Our findings will be used to establish current perceptions of the dashboards and prioritize gaps in knowledge, design, and use that can be considered for future dashboard applications.

## Methods

### Ethical Considerations

Advarra Inc, the HCS’s single institutional review board, approved the study protocol (Pro00038088). The HCS study is a registered trial (ClinicalTrials.gov NCT04111939).

### Research Setting

Data collection and analyses were conducted as part of the HCS, a 4-site, waitlisted community-level cluster-randomized trial seeking to significantly reduce opioid overdose deaths by implementing the CTH intervention in 67 communities across Kentucky, Massachusetts, New York, and Ohio [13,18]. For the first wave of the HCS, each site created interactive dashboards with community stakeholder input to support local data-driven decision-making using community-level metrics for 33 communities randomized to receive the intervention (ie, wave 1 communities) from January 2020 through June 2022 [14]. Our analysis involved all wave 1 communities: 8 communities from Kentucky, 8 communities from Massachusetts, 8 communities from New York, and 9 communities from Ohio.

### The CTH Dashboards

#### Common Components Across the Dashboards

More details about the cocreation of the dashboards and required core components can be found elsewhere [14]. Briefly, each site was responsible for developing a secure portal for each community receiving the CTH intervention. The portals contained downloadable intervention materials (eg, information on EBPs, community profiles, and community landscape data). Each portal had a CTH dashboard with data visualizations (eg, bar and line graphs and tables) displaying metrics related to the HCS’s primary outcome of reducing opioid deaths (eg, opioid overdose rates) and secondary outcomes connected to EBPs (eg, number of naloxone kits distributed and number of buprenorphine prescriptions filled).

#### Unique Components of the CTH Dashboards

Each site developed its CTH dashboard using different software, including Power BI (Microsoft Corp), Tableau (Salesforce Inc), SharePoint (Microsoft Corp), D3.js (Mike Bostock and Observable, Inc; Data-Driven Documents), Drupal (Drupal Community), and different types of visualizations, such as bar graphs, line plots, and tables with directional markers [14]. The 4 distinct dashboards displayed community-specific data that were accessible only to that community’s stakeholders. Data acquisition and display were limited by site-specific data use agreements, which informed the timing of the data (lags), data suppression, and granularity (eg, aggregation by state vs county), as well as the requirement of user and password-protected access in some cases.

### Study Design, Sampling, and Recruitment

**Figure 1:**
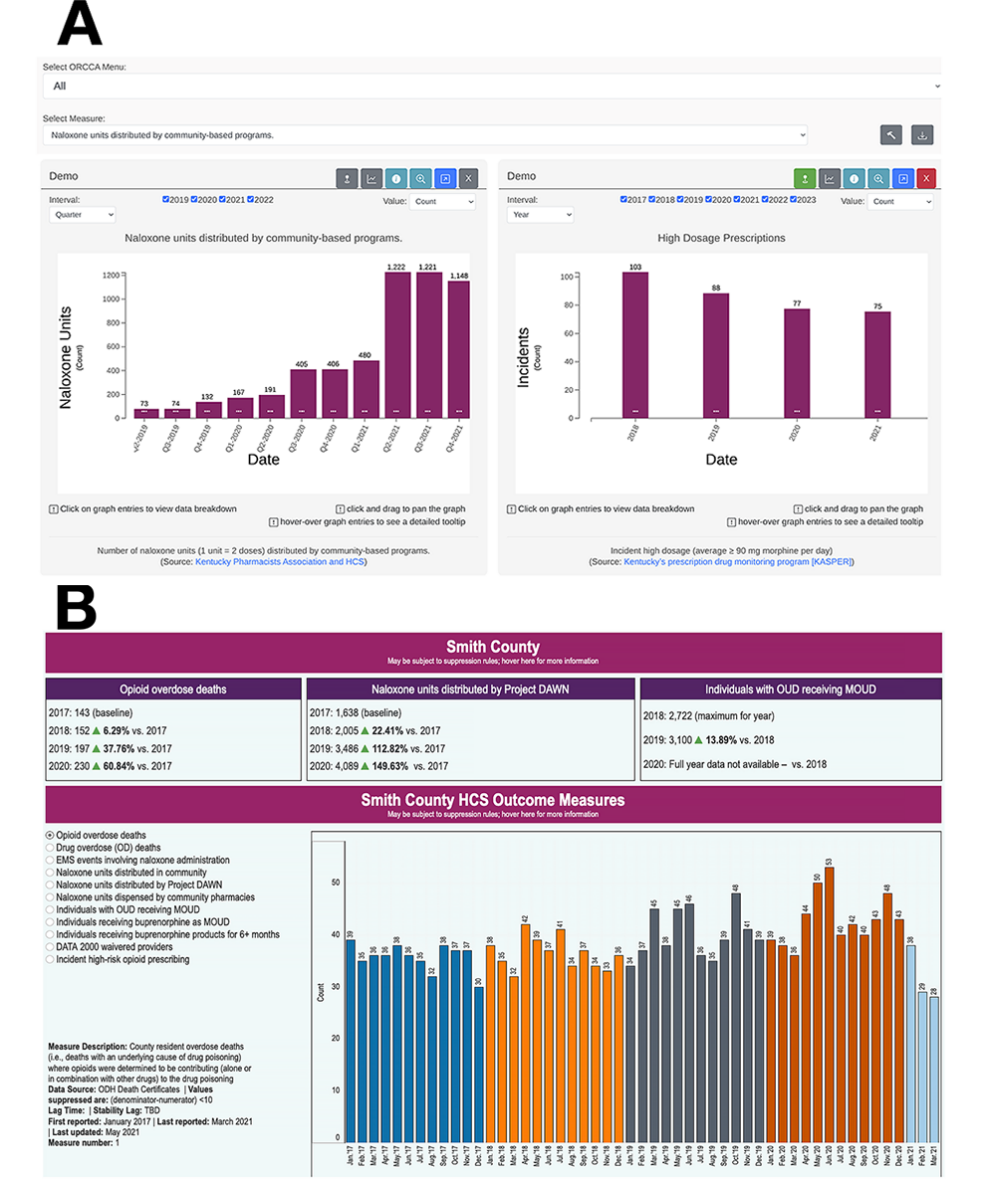
Mockups of the Communities That HEAL (CTH) dashboards: (A) CTH dashboard for Kentucky and (B) CTH dashboard for Ohio. Please note that any names of communities present are not real, and only synthetic data are used in the images. DAWN: deaths avoided with naloxone; EMS: emergency medical service; HCS: Healing Communities Study; MOUD: medication for opioid use disorder; OD: overdose; OUD: opioid use disorder; TBD: to be determined.

**Figure 2:**
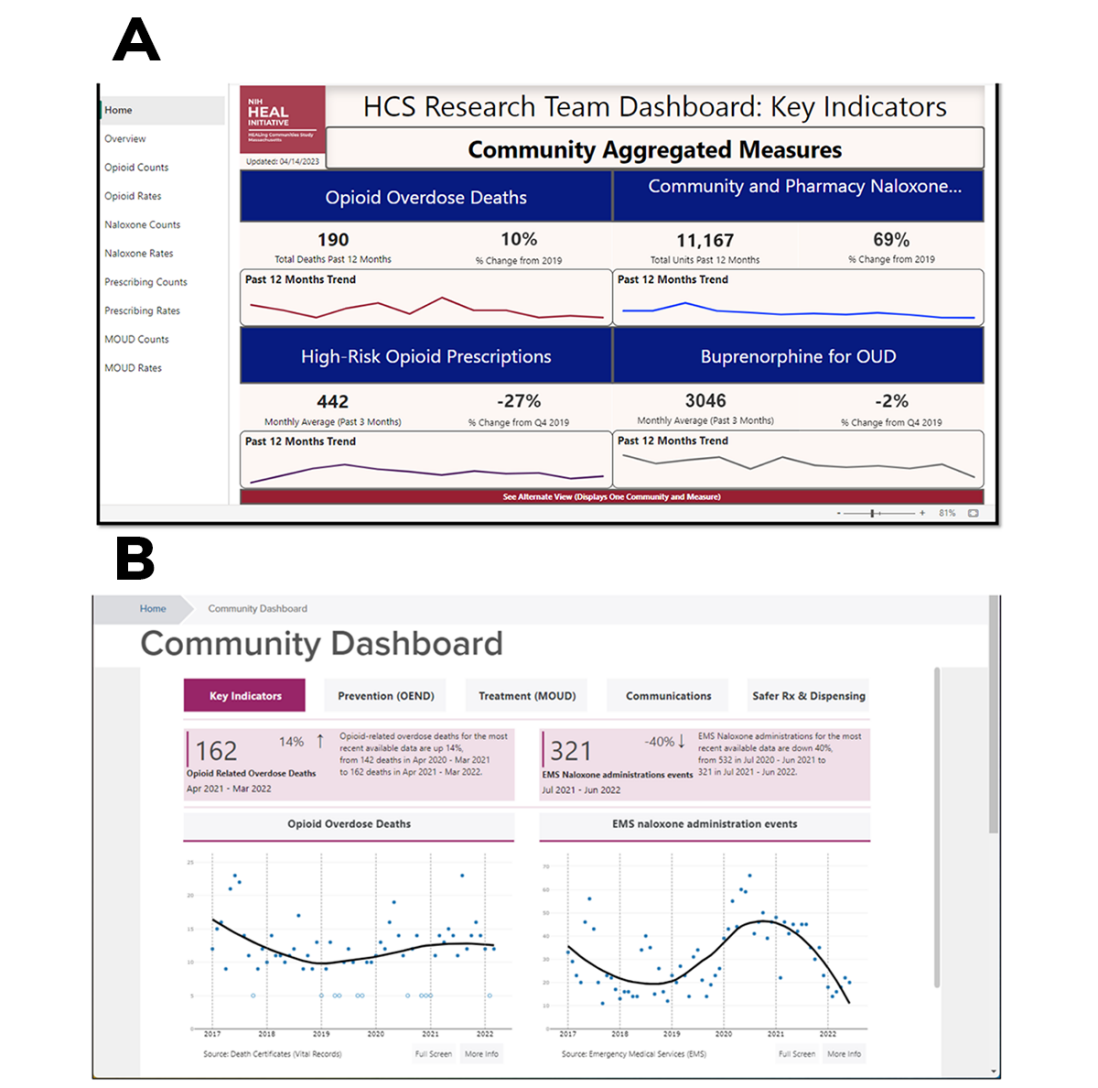
Mockups of the Communities That HEAL (CTH) dashboards: (A) CTH dashboard for Massachusetts and (B) CTH dashboard for New York. Please note that any names of communities present are not real, and only synthetic data are used in the images. EMS: emergency medical service; HCS: Healing Communities Study; HEAL: Helping to End Addiction Long-term; MOUD: medication treatment for opioid use disorder; NIH: National Institutes of Health; OUD: opioid use disorder.

Between June and July 2021, we conducted a mixed methods assessment of the use of the CTH dashboards. This period reflects the postimplementation phase of the second version of the cocreated dashboards (Figures 1 and 2) and was a stable time during which no fundamental revisions were made to the dashboards across the HCS. We administered the SUS to and conducted TAM-informed semistructured interviews with community stakeholders involved in the HCS. We collected sociodemographic data from participants to help characterize respondents. The interview data and SUS scores were concurrently examined to identify factors that influenced dashboard perceived use and usability.

Each site sampled and recruited participants. The sample was drawn from community stakeholders in the HCS communities who had a community portal account (ie, active users). Researchers from each site generated a roster of eligible participants from site server audit log files. Our research team worked with field staff to achieve a diverse participant pool of community stakeholder active users within each community. The sample included coalition members, including stakeholders in the HCS communities responsible for championing the use of a specific CTH intervention component, and HCS community staff who had the role of coordinating the use of data for decision-making. These active users were invited via email to participate in a group interview via Zoom (Zoom Communications, Inc) with up to 6 participants, although a few interviews were conducted individually due to scheduling challenges.

### Data Collection

Mixed methods evaluation data were collected by each site, which were then shared with the HCS data coordinating center (DCC) for analysis. Interviewers at each site first received common training on the research protocol, interview guide, and interviewing techniques. During the review, participants verbally provided consent and then were asked to individually fill out a short survey on REDCap [19]. The survey included sociodemographic questions and the SUS. After participants completed the survey, the research team displayed the site-specific CTH dashboard with synthetic community data to reorient participants to the dashboard used in their community. Next, the research team asked participants semistructured, open-ended questions based on the TAM dimensions of perceived usefulness, perceived ease of use, intention to use, contextual factors (Multimedia Appendix 1). Table 1 provides operational definitions for each of the TAM constructs used to develop our interview guide questions. We focused on 4 constructs to explore how the dashboards were used for community decision-making, their ease of use, the intent for future use, and the context around dashboard use. Our interviews lasted approximately 1 hour and were recorded and transcribed.

**Table 1:** Technology adoption model themes and operationalized definitions^a^.

Construct			Operationalized definition
**Perceived usefulness**			
	Description	Alignment of the dashboard and its functions with the community stakeholder’s expectations for goals and tasks	
	Benefits	How the dashboard positively influences the work and decision-making expectations of the community stakeholder	
	Drawbacks	How the dashboard did not meet the work and decision-making expectations of the community stakeholder	
**Perceived ease of use**			
	Description	Alignment of the technical functionality of the dashboard with the community stakeholder’s workflow (needs and desires)	
	Barriers	Specific challenges faced with using the portal	
	Facilitators	Specific resources needed to support the use of the portal	
**Intention to use**			
	Description	The willingness of the community stakeholder to use the dashboard in the future, even if modified slightly	
	Acceptance	The approachability of the dashboard as a technological tool to accomplish work	
	Preference	Specific improvements, changes, or recommendations to the dashboard	
**Contextual factors**			
	Description	Circumstances (eg, social, cultural, and historical circumstances) that influence a community stakeholder’s use of the dashboard	
	Community data orientation	Collective perceptions about how the community connects and works with data, including community data tools and approaches for decision-making	

^a^Definitions derived from Davis [16].

### Data Analysis

The validated SUS consisted of 10 five-point Likert scale questions. Each SUS item was scored (scale of 0 to 4), and their scores were summed and multiplied by 2.5 to assign a total score from 0 to 100 (lowest to highest usability). For the qualitative data, 1 research site constructed the codebook using an iterative constant comparative method [20]. Initially, the team created general codes using concepts from the TAM from the interview guide. Two researchers (RGO and YW) each coded an interview independently, compared coding, and discussed agreement and differences with a senior researcher (NF), creating a refined codebook with consensus codes and clarified definitions. The remaining interviews from this site were coded by these researchers, with review by the senior researcher. One researcher (RGO) working with the team inductively created subthemes for each TAM dimension, revised the codebook, and coded all the interviews from the site. The established codebook and coded interviews were then shared with the DCC. Two new coders from the DCC were trained by the experienced coders; each independently coded 6 test interviews and compared results in meetings with the experienced coders. Then, the DCC coders coded all remaining interviews (n=14) with weekly meetings to discuss differences and ensure alignment. The DCC coders then reviewed the original 7 interviews coded by the research site to confirm alignment across all data. After coding, the DCC conducted an exploratory analysis to better understand the emergent themes across different sites. Interview transcriptions were analyzed using NVivo (version 12; QSR International) [21].

The reporting of our qualitative methods and results adheres to the COREQ (Consolidated Criteria for Reporting Qualitative Research) guidelines (Multimedia Appendix 2; Table 1) [22]. Altogether, scientific rigor was supported by the use of participant IDs and labels to ensure data were appropriately associated with participants across communities; encouraging consistency in data collection and the fidelity of the interview guide through mentorship and weekly meetings to ensure agreement and the reliability of codes and results; discussions with various experts in the data visualization field during the development of themes; and triangulation (data from multiple HCS communities and diverse roles) and parallel data collection (SUS and TAM) to achieve theoretical sufficiency for themes and diverse representation across sites [23,24].

## Results

### Descriptive Summary of Survey Respondents and Interview Participants

A total of 159 individuals from across all HCS sites were invited to participate in our study. Of them, 62 (39%) individuals enrolled and participated in interviews. We conducted a total of 17 group interviews (with an average of 3 participants per group) and 10 individual interviews (with an average of 7 interviews per site). Community coalition members represented 56% (35/62) of interviewees, with the remaining 44% (27/62) comprising community staff hired by the HCS to work with their specific community coalition (Multimedia Appendix 2; Table 2). At least 1 person was interviewed from every community. Participants from all sites typically had a master’s degree, and a majority of participants from Ohio and Massachusetts were younger. Participants also typically identified as non-Hispanic White and female across sites (Multimedia Appendix 2; Table 3).

**Table 2:** Subthemes under perceived usefulness.

		Illustrative quotes
**Benefits**		
	Knowledge dissemination	“I think it’s a terrific dashboard. I love the way that the information is on there. I think it really captures every aspect of what we’re seeing on the street” (Ohio). “Our subcommittee particularly likes to see in the slides [from dashboard data] what’s going on month by month. We usually give them an overall of how many [naloxone kits] we’ve distributed in total, but they also like to see how we’re trending. So that’s basically been our main use of it” (Kentucky). “When you dive into the data dashboard, I would say that the biggest use for it has been just displaying any recent data we have pretty much on a quarterly basis, and that mainly happens at subgroup meetings” (Massachusetts).
	Decision-making tool	“[Deidentified person] can speak more to how data impacted our strategies. She was really helpful in the sense that like, she would give us the data, we would identify the issue, and then I would take that and talk to our partners to figure out how to move forward, but I wasn't the one really digesting it, pulling the stories from it” (New York). “Yes, I do believe that it definitely helped when it came to those decisions because it helped the entire coalition know certain areas that needed extra help and what kind of help that they needed. It also helped us form...helped us do the problem solving in the original start of this coalition” (Massachusetts). “Because it helped us choose...There’s 10 strategies that you could have chose, but by looking at the data that’s available on the dashboard, then you say, ‘All right, these are the ones that we need to tackle here in our community, because it’s right here in front of our face. These are the important ones’” (Kentucky).
	Access to more data	“So, we don’t see the practical application of this portal, but the information is on there. How to access it is, in my opinion, absolutely suited for any organization that would need that data that they don’t have access to normally” (Ohio). “But the Medicaid data, we never access that as the health department. I’m not entirely sure why but it’s a really helpful data source for us to be able to use because we serve a lot of, you know, medically underserved population. So, being able to have access to those numbers does help” (Ohio). “I got that from the dashboard, because I think that our sense was that there was more naloxone available than there probably was because there’s so many other sources now. I mean, it’s not just coming from harm reduction...And then we were trying to identify what was behind it because we were seeing that naloxone was a huge factor in deaths being down. I mean, that was something we had to really make sure was occurring. And if there was a distribution site, as it were, that was sort of flailing a little, we wanted to do something about that. So, that information came from the dashboard. I did not have a sense of that from the community” (Massachusetts). “[L]ike residents receiving buprenorphine. I don’t know where you’re getting that information, prescription drug monitoring programs. I wish we could get that like firsthand, but this is great to look at because I don’t get that data. And it’s current as of 2021” (New York).
**Drawbacks**		
	Time constraints	“[G]iven COVID and everything that happened we just didn’t have [data] available, and the timeline really just didn’t align with decision-making, and I think that is just a fundamental issue with the study, like we talked about, but I do think that that makes it challenging” (Massachusetts). “Particularly since we’ve been working out of the office, there’s greater demands of people’s time and lower bandwidth available” (Kentucky). “But this has also just been a unique for us Wave 1 folks, a unique time, given the pandemic and everything. We have so many other things too...that are not typical. That some of the HEALing [Communities] Study...has just been a little bit extra so it’s just been kind of a hard time...” (Ohio).
	Misalignment of the dashboard as a tool	“So, I would say that in [deidentifed community], a lot of it is redundant. They already have access to this data. It’s already in a location where they feel comfortable going and know how to go to. So, they don’t want to use something new. If they don’t have to” (Ohio). “I think, you know, had we had it earlier, I think it would have been easier to incorporate it into the coalitions. I think now it’s just trickier” (New York). “We did not use the data from the dashboard largely because, I think, everyone around our virtual table already knew all the data from our community dashboards and understood. Everyone is there because they work in this space, and so they understand exactly where the trends are, where the issues are, and so forth” (Kentucky).
	Disutility of data	“I think the factor is that the data is just so lagged, and the information that we need to work off of, we just need something way more current than what’s available...It’s just -- the data is just not very useful for us right now, unfortunately” (New York). “The problem is much bigger than what the numbers from [deidentified agency] can show us and so when we’re looking at it that way, it’s better to get that real time data from each other rather than rely on HCS to get old data that we’ve kind of already gone through” (Ohio). “Well, we’ve looked at the data, but I think we haven’t really used it, and the reason is two-fold. One, it’s too soon to be able to see much of an impact on the practices. We’re still rolling some of them out. And secondly, COVID, that has screwed up all the data, and we don’t, at the moment, have a good way to separate the impacts of the pandemic isolation and so forth from changes in practice in our community. Everyone right now is just kind of, with what’s going on, doing the best we can. We can’t read the data to detect impact of anything that we’re doing right now” (Kentucky).

**Table 3:** Subthemes under perceived ease of use.

		Illustrative quotes
**Barriers**		
	Access to dashboard	“This might be a little petty, but just the fact that I have to use a password to access it. I think especially people on our coalition, they may not write down their passwords that they use for [the HCS^a^] because it’s not their full-time job. So. if they forget the password, they’re less likely to go through the steps to retrieve it and get in there, so they may not use it as much if it were just open access” (Kentucky). “I remember my login info and its only because my computer remembers it. I think my username was given to me and it’s not what I normally use for things so I think if my computer didn’t hold my username and password, I would have had to fiddle with it every time I try to open it” (New York). “Another thing too, and this is my last thought is that it’s kind of odd who can have access and who cannot have access. It feels very gate kept in a way. And that’s going to be a barrier just anyway, so you know, we have our voting is set up for one vote per agency, which has led to just one representative per agency so that one representative for that agency is the only one who has access but their partner or a contact of theirs within their agency is the one emailing me for the information. They don’t have access to the dashboard. So that kind of does create a barrier as well and it’s definitely something that I don’t like about it. I don’t like there’s a password. I don’t like that you have to log in” (Ohio).
	Data manageability	“[Describing barriers and challenges] first of all it’s not being able to download the data but also not being able to compare two different data sets” (Ohio). “[I]nitially, they wanted us to have a couple slides and go over overdoses every month, and all of us were like, ‘What? There’s so many different things, and it looks insane. I don’t know how to explain that.’ Once I felt more comfortable with filtering things out to make it digestible, it’s been more useful. But initially, it was kind of crazy to look at” (Kentucky). “I might add that, as a CDM [community data manager] in a cluster community, these communities don’t often combine their data, so on the data dashboard for...those aggregate counts, or that aggregated together from each community, so it’s really hard to see what’s working, although we want to break silos. They don’t identify together, so I think that’s been a little bit hard, not to be able to see that separate breakdown” (Massachusetts).
	Unable to locate usable data	“I would say, a major barrier in [deidentifed community], as well, are the suppression rules, you know, because we have such a small population” (Ohio). “[I]t’s a rural community or communities, and so often our data is suppressed for the variables that require suppression. So, I think that just based on it’s great to remain confidentiality and is hard to understand if there’s an increase from one to four, et cetera, or zero to four. So, the suppression in rural communities is that point” (Massachusetts). “And so, we were anticipating and we were being told over and over again, that this data is coming, wait to see the dashboard, and then behind the scenes, we would get contacted and say, you guys got to push this dashboard, you guys got to keep pushing it, and so I’ll go on the dashboard and there’s nothing to push” (New York).
	Presentation of numbers and labels	“So, I’m assuming if any numerator is a one through 10 or a denominator’s one through 10, the data gets suppressed, but I guess I’m not 100% sure, and then...And then I noticed like on the overdose deaths for May of ‘21. There’s a zero value there. I don’t know if that’s like you know when there’s a zero on the dashboard. Are those true zeros or is it like a placeholder? Like do we really not have any overdose deaths in May at all. Which seems kind of, like it, you know not right, I guess” (Ohio). “Sometimes I wonder if, you know, the line across whatever the imputed data value [ie, values masked due to suppression rules] that is chosen for a particular measure, now that there’s the trend line, I wonder if just leaving off the imputed value all together, so it doesn’t show on the plot. Because I feel like if someone looks at it fast, they’re not looking at it close enough to be like, ‘Oh, why is March missing?’ They wouldn’t even notice that and that might clear up a bunch of confusion” (New York). “[On their naloxone administration event data] But yeah, it’s like on a five, it’s like five. And I remember somebody asking me like, ‘Why is it stuck at five instead of zero?’ I don’t know why. And I couldn't explain that. So, like, if I can't explain that, then like I don't -- like then they look at me, and, you know, whatever, we're all supposed to be experts and we all have degrees in-in, you know, in bio stats or data or whatever, we're experienced and if we can't explain that, then-then they question that” (New York). “My thing is super minor, and it’s a visual thing...On our specific dashboard, there’s the cover page, and the first one in the upper hand corner, it still says opioid overdose deaths, but if you click in it’s more like, I would say it’d be more accurate to describe it as opioid overdose events or related events, because it seems like, if someone were to click on it, they’d only be able to access the death data...When I present it, people were like, ‘Oh, I didn’t realize there was EMS data there too,’ so it’s totally a minor label thing” (Massachusetts).
**Facilitators**		
	Additional support	“I think, just like before maybe HEALing Communities leaves our county...It might be nice to like have a refresher to some of our stakeholders about the dashboard and what information is out there. You know, maybe like a you know presentation, or something and how to get access to it and that kind of thing. I know that was done a while back, because I feel like...I’m even a data person and I, you know still kind of struggle with the dashboard of HEALing Communities so and there’s a lot on there obviously that I didn’t even know about” (Ohio). “It may be helpful just for me to have some kind of training on how to put the data together though. Because I can go in there and just mess with everything. But looking at the community profile, the dashboards, and how to build those specific charts and things, I’m not sure exactly the functionality of it. So maybe just a video or just being able to ask questions or something like that would be pretty helpful” (Kentucky). “I was going to say for troubleshooting too, I think we’ve run into that challenge. Of course, coalition members will come to us saying they’ve run into an error or whatever the case may be, and it’s just challenging to triage some of that, to be like, ‘Hold on. Let me get in touch, try to figure out what’s going on, and then I’ll try to get back to you as soon as possible.’ And I think everyone in [Organization] has been really responsive” (Massachusetts).
	Data accessibility	“So, I do like that up at the top of the dashboard it has like where you are compared to 2017, it compares the various years to 2017 so that you see...the -- if it’s increasing or decreasing compared to that, like really steady fixed baseline. I also like that you can look at the data by month, by quarter, and by year, because I think that is also really helpful. Especially like with overdose deaths, where there can be some seasonality to that, it’s helpful to see that” (Ohio). “I like that you’re able to add more than one indicator to kind of look at how the trends have changed over the last couple of years and being able to put in more than one thing to get a graph and look at. Because it’s very helpful when it’s all in one place. Because I did a lot of data for a grant for that [deidentified] grant that I wrote a couple of years ago. And it was so hard to get all that data because you had to look at multiple different programs and now it’s just all on one program. So that’s really helpful” (Kentucky). “It’s quick, it’s like a bottom line, you know. If you, if you had to throw together a newsletter or a speech or a letter to the editor, you could, you know, you could glance over it and get some up to date, real time numbers, you know, for that area, pretty reliably” (New York).
	Navigability	“I think the dashboards, I think they’re beautiful, I think you guys did a really great job...I think it looks great, it just -- the data is just not very useful for us right now, unfortunately” (New York). “It’s very easy to use and I’m kind of a very visual person. So, for me, being able to look at graphs makes it a lot easier. And so, I use a lot of graphs...having those graphs provides me with a very quick and easy picture of ups and downs is really valuable” (Massachusetts). “I like the charts. I think that as someone who...I mean not only do I write grants and those charts are really helpful to stick in there for the reviewer, especially when you have something that you want to really emphasize. But also doing presentations and reports and things like that for other people, it really helps them to be able to see the trend and understand you know that. So, I just like the fact that it makes really nice, simple, pretty charts” (Ohio). “One of the things that I liked about it is being able to go in there and see the trends. It’s like it’s talking about pharmacy dispensing, and naloxone dispensing in the community, whether that’s trending up and down. I thought that was pretty helpful. It’s a few months behind, but an accurate view of what’s going on in the community right now” (Kentucky).

^a^HCS: Healing Communities Study.

### Perceived Usability and Acceptance of the CTH Dashboards

Of the 62 participants, 58 (94%; Kentucky: n=13, 21%; Massachusetts: n=14, 23%; New York: n=15, 24%; Ohio: n=16, 26%) completed the SUS survey. The grand mean for overall SUS scores across all HCS sites was 73 (SD 4.6), indicating that the CTH dashboards were within the 70th and 80th percentile of SUS responses (Multimedia Appendix 2; Table 4). This score is recognized in the literature as being within the acceptable range [25,26]. The overall SUS score for the New York dashboard was the highest (mean 77, SD 11.4) and akin to the SUS scores of Microsoft Word and Amazon. The overall SUS score for the Ohio dashboard was the lowest (mean 67, SD 12.3), which was similar to the usability of a GPS system. The overall SUS scores for the Kentucky and Massachusetts dashboards were 75.8 (SD 10.4) and 70.2 (SD 16.3), respectively.

**Figure 3:**
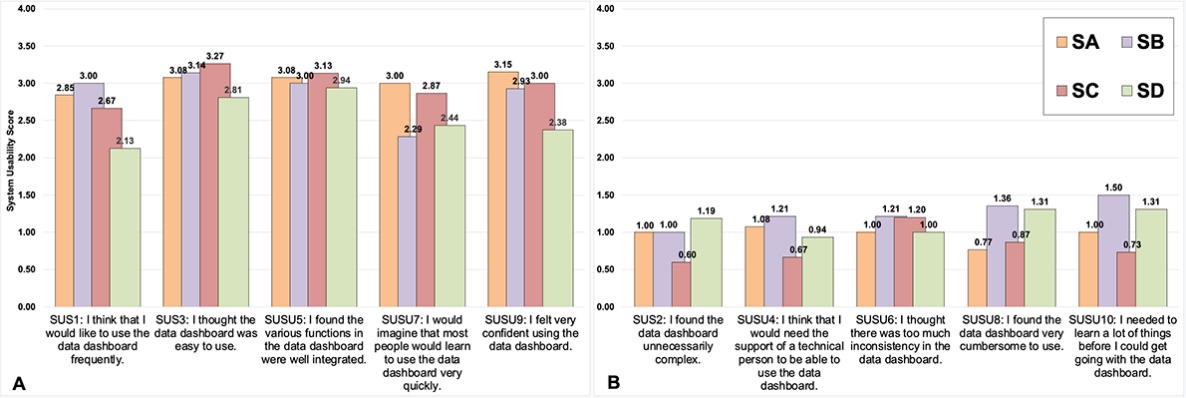
System Usability Scale (SUS) results by domain and study site (n=58). Raw SUS scores are presented. (A) SUS questions that were framed positively. (B) SUS questions that were framed negatively. Each SUS item is scored on a scale of 0 (strongly disagree) to 4 (strongly agree). A total of 4 participants did not respond to the SUS. SA: Kentucky; SB: Massachusetts; SC; New York; SD: Ohio.

Figure 3 illustrates the breakdown of each SUS domain and contrasts the average scores by participants from each HCS site. The New York dashboard scored the highest across most of the SUS domains. Although Ohio participants did not score the dashboard as favorably as participants from the other sites across many of the domains, it should be noted that the responses were generally positive. The discussion that follows expounds on these SUS scores, with additional support from the data from our TAM-informed interviews. We link specific SUS dimensions around usability with closely associated TAM themes on usefulness. This novel approach highlights potential drivers of SUS scores based on design considerations elucidated by a specific set of TAM themes and subthemes.

**Table 4:** Subthemes under intention to use.

		Illustrative quotes
**Acceptance**		
	Alternate data uses	“We are always, always, always pushing data driven decision-making with the coalitions and more often than not, the coalitions have a lack of data that they can specify down to a county or even a municipality. And so, having access to something like this...can potentially tap into the [deidentified community] data and be able to use that data to justify a program need or a grant...” (New York). “[W]e have opportunities...from new funding sources that we have never had before. So they’re coming towards us and kind of questioning where our gaps are and where we think that we could grow. So, I do think there’s things on the dashboard that we will probably be utilizing in the future” (Ohio). “I’m doing a study for my dissertation actually on...overdose deaths and things of that nature, and trauma, retention and treatment. All sorts of things. So, I’ll definitely be looking at the numbers that you guys have because they’re recent numbers. I know it’s valid data and things of that nature” (Kentucky).
	Future strategy monitoring and modification	“Maybe the dashboard will become more relevant as time goes on because it’ll be a longer period of time that we’ll be looking at and so we’ll have more time to reflect on and we’ll have a bigger window of time that we’re looking at” (Massachusetts). “So I think it’ll be even more important later on in the study after the community has been established, and the goals or your strategies have been in place for a while, and you can start seeing the effect” (Kentucky). “Maybe as things change in terms of opioid response, there might be a need to start looking at some other forms of data, but the data supplied now are okay, thank you” (New York).
	Use conditional on data changes	[When asked whether they would use the dashboard next year] “So I feel, I feel like if the data is, if I can track the same data on the dashboard that my DC [data coordinator] is giving to us, then yes” (Ohio). “[T]he reality is that the workload is very, very high and we’re frequently, and this won’t surprise you, we’re frequently coordinators and CEFs [community engagement facilitators] in a position of hurry up and do this today. And so it doesn’t allow for a lot of time for reflection and kind of that kind of thoughtful consideration...maybe it should be built into something a little bit more so that it is part of something” (Massachusetts). “I think it would be good for a community like the HEALing [Communities Study] to have that presence long term and having it be a problem-solving person means it could be one person serving multiple sites and not just ongoing doing something, but really targeted to needs. I think would be very helpful” (New York).
**Preference**		
	Content	“If we could break it down like [deidentied participant] said earlier by zip code that would be phenomenal because you know we wouldn’t have to wait for the meeting, we’d be able to see it anytime” (Ohio). “Well, I would love to see the information for the rest of the study in the community personally. I mean, I can understand why they limited it...I don’t mind comparing, you know, this one township to different counties and seeing how people are doing, you know...if the numbers showed something drastic, I would call over there and say, ‘What are you doing that we’re not doing?’” (New York) “One is that it feels a bit, I don’t know, not really, doesn’t get that accurate. Maybe it just can’t. Maybe there’s no way for it possibly to give an accurate illustration of the communities that it is supposed to represent, and maybe there’s just no way to do that. Maybe it’s just because I know the community so well, and maybe if somebody didn’t know the communities at all, they would look at that and say, ‘Wow, this is really helpful’” (Massachusetts).
	Function	“I just wish it was publicly available, because if I could send a link to somebody on my coalition there’s much better chance they’d be able to see it and access it” (Ohio). “It’s not accessible to general community or other groups that are not inside the [HCS^a^] structure. So, I guess there’s that piece, so thinking about that. Is there a purpose and a reason to make it public?” (Kentucky) “Something that has been frustrating for me, from some of the HCS data that we get is we get a lot of plots, but we never get the underlying data. And always what I want to do is be able to download it, download it into like visualization software that I use, add those like timeline components, and then present it in a way that I know my community will understand” (New York).
	Aesthetics	“My thing now is how can I make it into a Facebook post-type of thing to grab people’s attention; that we can start the conversation and engage them in the conversation. Having tools or in a format that we can say, ‘Let’s share this part and this part.’ Perhaps get some people engaged in talking about this issue or finding a champion about this issue. How can we make it usable like that?” (Kentucky) “I think it could be structured a little bit differently. You know specific to Healing Communities Study, you know, if we, if we really intended EBP [evidence-based practice] selection and monitoring for these tools, then I think they would have been they should be structured a little bit differently. The dashboard is getting there. I know we have like an MOUD view and a safer prescribing view, which I think is really helpful and you know if that was more like the primary way of looking at it. I think that would really increase its use for what we hope it would be used for” (Ohio).

^a^HCS: Healing Communities Study.

### Perceived Usefulness

Sites were generally positive in their reported perceptions about inconsistencies with the dashboards (SUS6); however, there was variability in the scores across states when it came to confidence in using the dashboards (SUS9). For example, participants from Ohio reported the least confidence, whereas participants from Kentucky scored the confidence domain the highest.

The alignment of the dashboards and community stakeholders’ expectations may have influenced perceptions of confidence in using the dashboards and consistency in using the dashboards. On the basis of the TAM-informed interviews, 3 subthemes emerged regarding the benefits of using the CTH dashboards: *knowledge dissemination* (the dashboard was used to increase the awareness of activities in a community [eg, sharing data and validating existing assumptions]); *decision-making tools* (the dashboard was used for choosing EBPs in a community and evaluating EBPs strategies); and *access to more data* (broader and granular dashboard data about a community were available). In contrast, 3 subthemes emerged regarding the drawbacks of using the CTH dashboards: *time constraints* (the use of the dashboard felt time intensive, and other priorities distracted from its use); *misalignment of dashboard as a tool* (the use of the dashboard was not aligned with the HCS’s goals and workflow); and *disutility of data* (dashboard data were not translatable to anything actionable). Table 2 provides illustrative quotes from the interviews for each of the primary themes under perceived usefulness.

### Perceived Ease of Use

Around integration (SUS5), perceptions among all participants were generally positive. Participants from Ohio did not respond as positively about ease of use and complexity of use (SUS2 and SUS3). Participants from Massachusetts, New York, and Kentucky scored the dashboards higher in these areas. Participants from Ohio and Massachusetts also scored training, support, and the cumbersomeness of the system (SUS4, SUS7, and SUS8) less favorably.

The alignment of the technical functionality of the dashboards with users’ workflows may be linked to the users’ perceptions of the dashboards’ complexity and ease of use and the need for training and support. Our TAM-informed interviews revealed 4 subthemes around barriers to use: *access to dashboard* (participants faced challenges with accessing the dashboard); *data manageability* (accessing the data users needed was cumbersome); *unable to locate usable data* (data in the dashboard were not easy to find because of the lack of availability [due to lags in reporting or suppression rules] or because of navigation issues on the dashboard); and *presentation of numbers and labels* (the data were displayed in ways that made their use difficult for participants). However, we discovered 3 subthemes that facilitated the use of the dashboards: *additional support* (training or information that informed the use of the dashboard or data); *data accessibility* (the dashboard was useful because of aspects of the technology or the way it is displayed); and *navigability* (different ways in which the dashboard was easy to use and navigate and aspects of the dashboard that could be changed to make it better). Table 3 provides illustrative quotes for each of the primary themes under perceived ease of use.

### Intention to Use

Scores of the SUS items on the frequency of planned use and the knowledge needed to use the dashboard (SUS1 and SUS10) were equivocal and lower across all the sites. Participants from Massachusetts reported that they wanted to use the dashboard frequently, but they needed to learn more about how to use it. Participants from Ohio provided the lowest score for the SUS item on the frequency of planned use of the dashboard and, similar to their Massachusetts counterparts, indicated the need to learn more about how to use the dashboard.

The willingness of community stakeholders to use the dashboard in the future may influence their desire to use the dashboard frequently and learn how to use it. During the TAM-informed interviews, participants provided several recommendations for improving the dashboard that could promote future use. A total of 3 subthemes emerged regarding the acceptance of the dashboards in the future: *alternate data uses* (data on the dashboard will be useful but for purposes not initially part of the focus of the HCS); *future strategy monitoring and modification* (the dashboard will be used as intended for HCS purposes, including for EBP strategy monitoring and data-driven decision-making about these strategies); and *use conditional on data changes* (the dashboard will be useful if some conditions are met [to help with its use]). In addition, 3 subthemes emerged regarding preferences with desirable use over time: *content* (participants desired changes to the data on the dashboard, including the provision of different data, to improve the usability of the data and their interpretation); *function* (participants desired additional tools or different ways to navigate the dashboard and use data); and *esthetics* (participants described ways in which the appearance of the dashboard could be improved to effectively communicate information). Table 4 provides illustrative quotes on recommendations for each of the primary themes under intention to use.

### Contextual Factors

Collective circumstances may influence the dashboard’s perceived usability and use. A total of 2 subthemes emerged regarding how the community connects and works with data (community data orientation): *comfort with data* (collective perceptions of the comfort with using tools such as dashboards that contain data to make decisions); *established tools and approaches* (perceptions that ranged from those of human-centered data sources [eg, existing relationship with a coroner’s office] to technology-based sources that were community preestablished substitutes to the HCS dashboard). Table 5 provides exemplary quotes for each of the primary themes under the community context that illustrate how dashboards and their use may have been perceived when implemented.

**Table 5:** Subthemes under contextual factors.

Community data orientation	Illustrative quotes
Comfort with data	“[T]he treatment providers, they’re not necessarily data people, but there’s some of us that are looking at it and using it. I guess that’s all that matters in the long run. That’s something that we’re always going to be dealing with the people in the coalition that we have” (Massachusetts). “I think it goes back to who your audience is...If you’re looking to speak to people who, you know, I kind of consider myself a middle person for that aspect, right, so if you’re trying to speak to community members who have no data background and things like that, they’re -- first of all, they’re not even going to notice that [a date] is missing...” (New York). “[S]o we are planning on either in our August or September drug coalition meeting kind of...showing people how it works, showing people the data that’s in there, and hopefully getting people a little bit more oriented towards...looking at that dashboard to see if what we’re doing is making a difference, because I don’t think that we are, as a coalition I don’t think we are data oriented and enough really if that makes sense” (Ohio).
Established tools and approaches	“I mean, I think the primary source or the primary...way that data is distributed in [the community] is really just through personal connections...I mean you know I think every coalition meeting we’ve had our health commissioner comes and has the...harm reduction clinic numbers, you know written down on the back of an envelope or a napkin or something and that was you know the you know the organizations are generally very good about sharing that individual level data...You know, to help, to help people” (Ohio). “So, they’ve got their ODMAP [Overdose Detection Mapping Application Program] data that comes in through HIDTA [high intensity drug trafficking areas] and the police departments, and then they have the [Department of Health] data that shows how many Narcan saves happened and how many overdoses occurred. And then on top of that, you have things like coroner’s reports and coroner responses to actual deaths” (New York). “I would say there’s a couple of places we’ll look depending on what it is. We use the KIPRC site a lot, Kentucky Injury Prevention. We use that depending on what they have. We also keep a data dashboard from the Health Department’s perspective on substance use and opioid disorder. So, we have a dashboard on ours that looks at things. We do a daily dashboard on overdose visits to the ED and EMS runs and things like that...Periodically, we might get things from the Office of Drug Control Policy, the state. We also have a local Office of Drug Control Policy, which pulls some local data for all of our counties as well. So, I would say those are probably our main sources of substance use data” (Kentucky).

## Discussion

### Principal Findings

Our assessment indicated that the community stakeholders in the HCS found the CTH dashboards to be usable, as measured by the SUS, and easy to use and understand, as indicated by the themes identified through our TAM-informed interviews. Some respondents indicated the usefulness of the dashboards, with many indicating areas for improvement. From these findings, we have synthesized and prioritized the following gaps and lessons learned for future consideration, which are generalizable to community stakeholders engaged in dashboard use. In the spirit of Chen and Floridi [27], our lessons learned are specifically about different visualization pathways for use in dashboards among community stakeholders. Prior research has identified similar practices as supportive of the effective use (eg, greater engagement, cognitive alignment between end users, and decision aids) of dashboards. The lessons learned that we describe subsequently may help researchers design higher-fidelity dashboards that future scientific studies should consider when developing similar interventions and more general tools that integrate data visualizations for use among community stakeholders in community-oriented studies.

### Prioritized Gaps in and Lessons Learned About Designing Dashboards for Community Stakeholders

#### Prioritized Gap: Cognitive Dissonance With the Dashboards

Many of the community stakeholders already had frontline knowledge of their community opioid crisis and its complexities. Hence, some community stakeholders may have seen the dashboards as tools simply providing hard data, which were secondary to their lived experiences within their communities and knowledge about contextual nuances with these communities. There may have been, arguably, a cognitive disconnect between what the community stakeholder expected from a dashboard and what the dashboard provided. This disconnect may have been exacerbated by factors such as adherence to the main HCS research protocol and purpose (eg, a brief timeline for development and programming before deployment and the prohibition of data downloads from visualization), use of suppression rules that masked values and contributed to the spareness of data, lags in metrics due to reporting, and limited connections of the data with local resources for community stakeholders to act upon. Other challenges included balancing the need for “real-time” data with the validity of these data due to retrospective corrections.

#### Lesson 1: Use Storytelling via Dashboards

Cognitive and information science theories suggest the importance of aligning information representation formats provided by decision aids with mental representations required for tasks and cognitive styles of individuals [28-30]. We propose storytelling via dashboards as an effective, historically validated [31] approach to achieving this alignment. At a minimum, this involves providing basic data summaries in plain English, as done by the New York HCS site, and mixing and matching measures in graphs to allow the illustration of specific points, as adopted by the Ohio and Kentucky sites. Richer storytelling may involve multiple iterations or cocreations with community stakeholders that help craft the right set of qualitative contexts and information that situates quantitative data to evoke understandings that fit with internal mental models of the task and lived experiences [32,33]. Powerful stories could provide community stakeholders with structured templates to analyze, justify, and communicate data. Our findings suggested that there were indeed community stakeholders who were not comfortable with using data; individuals with such predispositions could find data presented as stories more meaningful, especially during decision-making processes.

#### Lesson 2: Link Actionable Insights to Useful Resources

Community stakeholders may require additional support with using insights garnered from dashboards. Dashboards can be seen as a mediator between data and a call to action to address specific issues. Moreover, dashboards can present structured sets of actions a community stakeholder can undertake, also known as an actionable impetus [34], as guidance on addressing a problem identified with the data [35-37]. For example, some community stakeholders in the HCS would have appreciated a set of recommendations for EBPs or resources within a local neighborhood that a coalition could have acted upon, given an outbreak of opioid deaths. Design considerations could have made affordances (eg, alerts in the user interface) to create an impetus for action, which has been described as an “actionable data dashboard” [34]. Such actionable dashboards could provide important links to specific issues with the opioid crisis, such as the linkage of distribution centers for naloxone kits to community hot spots where there is unequal access to these kits based on factors such as race and ethnicity [38].

#### Prioritized Gap: Gatekeeping of the Data and Dashboards

Access to the dashboards was a noteworthy barrier under perceived ease of use. Some study participants indicated that this barrier hindered the use of data to facilitate decision-making among community members. Dashboard requirements limiting access to certain stakeholders was due to study protocol requirements to prevent communities from benchmarking data across communities. However, the user and password verification requirements were seen as inconvenient, and access control was seen as a form of gatekeeping that limited information sharing with key stakeholders who did not have dashboard access and were critical to decision-making in communities.

#### Lesson 3: Use Processes and Tools That Promote Access and Sharing

Arguments to enhance the use of data included approaches that facilitate human-human interactions in the sensemaking of data within dashboards and visualizations [3,39,40]. In addition to storytelling, other practices include improving how study insights are shared with communities and how communities are encouraged to share insights; permissible sharing includes dashboard export features or providing embedded codes so that data or narratives can be used in media, reports, presentations, websites, and social media content [41].

#### Prioritized Gap: Low Engagement With the Dashboards

Several themes (eg, data manageability, additional support, use conditional on data changes, and aesthetics) highlighted additional challenges to perceived ease of use and intention to use that could be represented by a broader concept used in the technological literature known as digital engagement [42-44]. The HCS missed opportunities to promote community stakeholder engagement with the CTH dashboards through which enhancements could have been made in areas such as dashboard training and clearer protocols around how community stakeholders used the dashboards during coalition meetings.

#### Lesson 4: Use a Multisector and Interdisciplinary Approach to Understand and Improve Engagement With Data Visualizations

The use of audit log file data or eye tracking to monitor and characterize use and user preferences (eg, line chart vs bar graphs) can inform design decisions and tailored interventions to foster behavioral changes that support the effective use of data among community stakeholders [44-46]. Our study, while recognizing early on that log files can support engagement, faced challenges with implementing a standard data model to track visual preferences and visit frequencies among community stakeholders, a decision that could have supported systematic transformations to the dashboards. In addition, our findings demonstrated that the combined use of SUS scores and TAM-informed interview data was beneficial in obtaining a comprehensive perspective on how engagement, via usability, can be transformed. For example, prior research has indicated that barriers in usability may have been overlooked if only measured by a single approach, such as exclusively using the SUS [47].

The use of innovative training (eg, dashboard navigators and indexed videos) and workflow changes (eg, starting meetings with referencing insights from a dashboard) are 2 other practice-based examples that can foster engagement among community stakeholders by supporting the realignment of existing mental models and the building of new ones with visual representations to achieve specific tasks [48,49]. For example, the Massachusetts HCS site adopted “community data walks,” in which expert dashboard users provided active demonstrations to community stakeholders on how to use the CTH dashboards.

Our findings suggest that the fidelity of the cocreation process to develop the CTH dashboards had gaps. The HCS is one of the first large-scale applications of the cocreation process to design dashboards within the community context. The pragmatic, multisetting nature of the study reflects challenges to balancing a research agenda with the expectations of diverse communities. Interventions and strategies (eg, multiple plan-do-study-act cycles during the cocreation process) that can address gaps in fidelity in using cocreation could be adopted to ensure that the final outputs are indeed relevant to end users’ work and promotes higher engagement. There were no “checklists” to support our implementation at this level of complexity, and we hope that the lessons we learned establish the foundations for such an approach for future endeavors.

### Limitations

Our definition of community stakeholders may be idiosyncratic to our study and parent study research protocols. However, the 4-site design provides a diverse sample of individuals who represent different professional roles and social and demographic groups; the samples were not homogeneous across sites, including in terms of technology literacy, which was not assessed yet could shape end users’ experience with the dashboards [48]. The COVID-19 pandemic may have influenced perceptions of use of and intentions to use the CTH dashboards, given pandemic-driven competing demands, technology-mediated workflow, etc, experienced by the respondents. This may have resulted in conservative perceptions of dashboard usability; however, the public health domain is generally faced with competing demands and emerging situations, which might mitigate such notions. Our research study was relatively large in scope; hence, ensuring the fidelity of our assessment in general and the interviews specifically was challenging. However, as noted in the Methods section, we worked closely across sites to maintain rigor from study inception. We acknowledge that although the SUS is a validated tool, it may have lacked specificity for assessing CTH dashboards. Other TAM constructs (eg, external environment) and more updated versions of the TAM exist [50,51]. However, one of our original goals was to build a framework for dashboard use among community stakeholders; therefore, we chose to use a fundamental version of the TAM that had both the critical constructs for our focus and did not have other constructs that may have imposed a priori assumptions on dashboard usability from other fields and studies.

### Conclusions

Data dashboards can be an evidence-based approach to support community-based public health decision-making. These technological interventions to visualize and interact with data can support transformations in community health, including during public health crises, such as the COVID-19 pandemic and the opioid epidemic. Our study is a novel assessment using both the SUS and TAM to examine the usability of dashboards among community stakeholders. Participants provided multifaceted perspectives on the usability of the CTH dashboards and their intention to use these dashboards. On the basis of our findings, we presented important gaps that motivated the consolidation of lessons learned regarding dashboard use among community stakeholders.

## Appendix

Multimedia Appendix 1 Semistructured group interview guide on dashboards.

Multimedia Appendix 2 Supplementary tables with additional data.

## Abbreviations

COREQ: Consolidated Criteria for Reporting Qualitative Research
CTH: Communities That HEAL
DCC: data coordinating center
EBP: evidence-based practice
HCS: Healing Communities Study
SUS: System Usability Scale
TAM: technology adoption model
