# Supplementary material for: Lessons Learned From Developing Dashboards to Support Decision-Making for Community Opioid Response by Community Stakeholders: Mixed Methods and Multisite Study
Source: JMIR Hum Factors. 2024 Sep 9;11:e51525. doi: 10.2196/51525 (PMC11420584; doi:10.2196/51525)
Supplement: Multimedia Appendix 1 [file humanfactors_v11i1e51525_app1.docx]

# **Supplement File 1**

**Semi-Structured Group Interview Guide on Dashboards**

Created April 5, 2021

Everyone here may have different experiences using the dashboards, and we are interested in hearing about all of these experiences. Some of you may have used the dashboard quite a bit and some of you may not have used the dashboard much or at all. To make sure everyone is discussing the same thing, I will show an example of a HCS dashboard that is not from your community and does not have real data but is used to remind you of what your community dashboard looks like.

The questions I will ask you are about the dashboard for your community.

*[DISPLAY/PROVIDE SAMPLE DASHBOARD (THAT USES DUMMY DATA). PAGE/CLICK THROUGH METRICS, AREAS, AND FEATURES AS APPROPRIATE TO PROMPT, PROBE, CONFIRM, ETC.]*

- Thinking of the dashboard for your community, can you explain the ways, if any, that you have used the data dashboard?
  - [POSSIBLE PROBES]: What do you like about the dashboards? What are some features or aspects that you think other stakeholders and communities would really like (i.e., “delightful” features)?
  - [POSSIBLE PROBES]: What don’t you like about the dashboards?
  - [POSSIBLE PROBES]: What specific challenges or barriers have you faced when using the dashboard
  - [PROBES ON EBP SELECTION]: Thinking of the time you’re your coalition was selecting evidence-based practices from the ORCCA menu, how did you use data from the dashboards to inform those decisions? If you didn’t use the data, why not?
  - [PROBES ON MONITORING DATA] In the time since the evidence-based practices were selected and initially deployed, how have you used the dashboards to monitor or evaluate the evidence-based practices or informed changes to the practices in your community? If you haven’t used the data, why not?
- In the next year, do you see yourself using the dashboard and how would you use it?
- Besides the HCS dashboard, when you need data, how do you get the information you need?
  - - [POSSIBLE PROBES]: What alternative resources for data do you use? What makes these resources useful for you? What is missing from the dashboard or would make it more useful for you?

**GROUP INTERVIEW CLOSURE**

- Do you have any other thoughts about how the data dashboard could be used to address the opioid crisis in your community?

Thank you for your time and participation!
