# Supplementary material for: Lessons Learned From Developing Dashboards to Support Decision-Making for Community Opioid Response by Community Stakeholders: Mixed Methods and Multisite Study
Source: JMIR Hum Factors. 2024 Sep 9;11:e51525. doi: 10.2196/51525 (PMC11420584; doi:10.2196/51525)
Supplement: Multimedia Appendix 2 [file humanfactors_v11i1e51525_app2.docx]

# **Supplementary File 2**

Table of Contents

[Supplementary File 2 1](#_Toc122511342)

[Supplemental Table 1. Summary of COREQ guidelines and study activities 2](#_Toc122511343)

[Supplemental Table 2. Summary of group interview participants and representation 4](#_Toc122511344)

[Supplemental Table 3. Demographics of community data dashboards survey respondents, by research site (n=62) 4](#_Toc122511345)

[Supplemental Table 4. Summary of community data dashboards SUS survey responses, by research site (n=58) 5](#_Toc122511346)

| **Supplemental Table 1.** Summary of COREQ guidelines and study activities | | |
| --- | --- | --- |
| **No. Item** | **Guide questions/description** | **Reported** |
| **Domain 1: Research team and reﬂexivity** |  |  |
| *Personal Characteristics* |  |  |
| 1. Interviewer/facilitator | Which author/s conducted the interview or focus group? | KY: Elizabeth Larimore, Ron Langley  MA: Peter Balvanz, Sarah Kosakowski  NY: Elwin Wu, Erin Kim, Anna Weiskopf  OH: Naleef Fareed, Ramona Olvera, Rachel Chase |
| 2. Credentials | What were the researcher’s credentials? e.g. PhD, MD | Master or PhD  EL, PB, SK, EK, AW – Masters education  RL, EW, NF, RO, RC - PhD |
| 3. Occupation | What was their occupation at the time of the study? | HCS Research Staff |
| 4. Gender | Was the researcher male or female? | RL, PB, EW, NF – male  EL, SK, EK, AW, RO, RC – female |
| 5. Experience and training | What experience or training did the researcher have? | Diversity of qualitative interviewing experience though all staff were trained on the instrument (by I8, I9, and I10). All staff had experience with the HCS study. |
| *Relationship with participants* |  |  |
| 6. Relationship established | Was a relationship established prior to study commencement? | With community coalition members: Interviewers were familiar with the communities and may have been on meetings with the participants;  HCS staff – The interviewers and interviewees worked with each other, but direct relationship varied based on site. |
| 7. Participant knowledge of the interviewer | What did the participants know about the researcher? e.g. personal goals, reasons for doing the research | All participants were familiar with the aims of the overall HCS study and had participated in multiple aspects of the study. The goals of this specific research study were clarified to the participants during the introduction and consent process. |
| 8. Interviewer characteristics | What characteristics were reported about the inter viewer/facilitator? e.g. Bias, assumptions, reasons and interests in the research topic | EW, NF, RL, PB all had roles in developing the first dashboards and worked closely with the Portal WG. |
| **Domain 2: study design** |  |  |
| *Theoretical framework* |  |  |
| 9. Methodological orientation and Theory | What methodological orientation was stated to underpin the study? e.g. grounded theory, discourse analysis, ethnography, phenomenology, content analysis | Technology Acceptance Model (TAM); general qualitative interviewing (discourse analysis). |
| *Participant selection* |  |  |
| 10. Sampling | How were participants selected? e.g. purposive, convenience, consecutive, snowball | Purposive – Sample from those who had access (HCS approved log-in) to the dashboards, had accessed the dashboard. |
| 11. Method of approach | How were participants approached? e.g. face-to-face, telephone, mail, email | Email recruitment of those already part of HCS. |
| 12. Sample size | How many participants were in the study? | 62 (27 staff/25 coalition members) |
| 13. Non-participation | How many people refused to participate or dropped out? Reasons? | 97 (5 staff/ 92 coalition members) did not participate in interviews, either because of refusal or because of site reached targeted sample participation. |
| *Setting* |  |  |
| 14. Setting of data collection | Where was the data collected? e.g. home, clinic, workplace | Virtually (Zoom) |
| 15. Presence of non-participants | Was anyone else present besides the participants and researchers? | Multiple participants attended group interview. Because of the nature of Zoom, it is unclear if others were present off camera. |
| 16. Description of sample | What are the important characteristics of the sample? e.g. demographic data, date | Staff – coalition members; Rural – urban community distribution; high – low dashboard usage. |
| *Data collection* |  |  |
| 17. Interview guide | Were questions, prompts, guides provided by the authors? Was it pilot tested? | Interview guide approved by portal workgroup; pilot of guide completed as part of the training session. |
| 18. Repeat interviews | Were repeat interviews carried out? If yes, how many? | No |
| 19. Audio/visual recording | Did the research use audio or visual recording to collect the data? | Audio Recording of Zoom interviews which were transcribed. |
| 20. Field notes | Were ﬁeld notes made during and/or after the interview or focus group? | Interviewers may have taken notes, but these were not part of the data. |
| 21. Duration | What was the duration of the interviews or focus group? | Approximately 60 minutes. |
| 22. Data saturation | Was data saturation discussed? | We focused initially on cross site theoretical sufficiency for parent codes (discussed weekly at national meetings); then the lead analytical site focused on theoretical sufficiency at sub-theme level; and finally discussed and validated sufficiency of sub-themes with DCC during the cross site assessment. |
| 23. Transcripts returned | Were transcripts returned to participants for comment and/or correction? | No |
| **Domain 3: analysis and ﬁndings** |  |  |
| *Data analysis* |  |  |
| 24. Number of data coders | How many data coders coded the data? | Lead analysis site: 2 coder, 1 senior researcher  DCC: 2 coders, 1 senior researcher |
| 25. Description of the coding tree | Did authors provide a description of the coding tree? | Coding tree was developed by lead analysis site research team. |
| 26. Derivation of themes | Were themes identiﬁed in advance or derived from the data? | Parent codes were based on TAM and child codes were developed inductively from the data. |
| 27. Software | What software, if applicable, was used to manage the data? | NVivo 12 |
| 28. Participant checking | Did participants provide feedback on the ﬁndings? | No |
| *Reporting* |  |  |
| 29. Quotations presented | Were participant quotations presented to illustrate the themes/ﬁndings? Was each quotation identiﬁed? e.g. participant number | See results – At least 3 quotations were found for each child code; participants identified by high-low use and state. |
| 30. Data and ﬁndings consistent | Was there consistency between the data presented and the ﬁndings? | Yes, and reflected in the discussion section. |
| 31. Clarity of major themes | Were major themes clearly presented in the ﬁndings? | Major themes connect to the TAM (pre-existing themes) and are presented in the results. |
| 32. Clarity of minor themes | Is there a description of diverse cases or discussion of minor themes? | Discussion of the minor themes are presented in the paper. |

Note: Consolidated criteria for reporting qualitative studies (COREQ): 32-item checklist developed from Tong, A., Sainsbury, P., & Craig, J. (2007). Consolidated criteria for reporting qualitative research (COREQ): a 32-item checklist for interviews and focus groups. International journal for quality in health care, 19(6), 349-357.

| **Supplemental Table 2.** Summary of group interview participants and representation | | | | | | | | |
| --- | --- | --- | --- | --- | --- | --- | --- | --- |
| **Site** | **Staff Invited** | **Coalition Members Invited** | **Interviewed Staff n (% of invited)** | **Interviewed Coalition Members n (% of invited)** | **Total Interviewed n (% of invited)** | **Urban Interviewed n (% of total interviewed)** | **Coalition Members Interviewed n (% of total interviewed)** | **Number of Group Interviews** |
| **KY** | 6 | 34 | 5 (83) | 8 (24) | 13 (33) | 7 (54) | 8 (62) | 7 |
| **MA** | 11 | 16 | 10 (91) | 5 (31) | 15 (56) | 10 (67) | 5 (33) | 6 |
| **NY** | 8 | 17 | 8 (100) | 10 (59) | 18 (72) | 13 (72) | 10 (56) | 7 |
| **OH** | 7 | 60 | 4 (57) | 12 (20) | 16 (24) | 8 (50) | 12 (75) | 7 |
| **Total** | 32 | 127 | 27 (84) | 35 (28) | 62 (29) | 38 (61) | 35 (56) | 27 |

| **Supplemental Table 3.** Demographics of community data dashboards survey respondents, by research site (n=62) | | | | | | |
| --- | --- | --- | --- | --- | --- | --- |
| **Characteristic** | | **KY n (%)** | **MA n (%)** | **NY n (%)** | **OH n (%)** | **Total n (%)** |
| **Age** | 18-34 Years | 3 (23.1) | 7 (50.0) | 4 (26.7) | 6 (37.5) | 20 (34.5) |
|  | 35-49 Years | 6 (46.2) | 3 (21.4) | 2 (13.3) | 7 (43.8) | 18 (31.0) |
|  | 50-64 Years | 4 (30.8) | 3 (21.4) | 7 (46.7) | 3 (18.8) | 17 (29.3) |
|  | 65-74 Years | 0 (0.0) | 1 (7.1) | 2 (13.3) | 0 (0.0) | 3 (5.2) |
| **Education** | Associate Degree | 1 (7.7) | 0 (0.0) | 0 (0.0) | 1 (6.3) | 2 (3.4) |
|  | Bachelor's Degree | 2 (15.4) | 3 (21.4) | 3 (20.0) | 2 (12.5) | 10 (17.2) |
|  | Doctorate | 1 (7.7) | 0 (0.0) | 2 (13.3) | 0 (0.0) | 3 (5.2) |
|  | HS Degree or Equivalent | 0 (0.0) | 0 (0.0) | 0 (0.0) | 0 (0.0) | 0 (0.0) |
|  | Less Than HS Diploma | 0 (0.0) | 0 (0.0) | 0 (0.0) | 0 (0.0) | 0 (0.0) |
|  | Master's Degree | 8 (61.5) | 8 (57.1) | 9 (60.0) | 10 (62.5) | 35 (81.4) |
|  | Prefer Not to Answer | 0 (0.0) | 1 (7.1) | 0 (0.0) | 0 (0.0) | 1 (1.7) |
|  | Professional Degree | 1 (7.7) | 0 (0.0) | 1 (6.7) | 0 (0.0) | 2 (3.4) |
|  | Some College | 0 (0.0) | 2 (14.3) | 0 (0.0) | 3 (18.8) | 5 (8.6) |
| **Ethnicity** | Hispanic/Latino | 0 (0.0) | 0 (0.0) | 1 (6.7) | 1 (6.3) | 2 (1.7) |
|  | Non-Hispanic/Latino | 13 (100.0) | 14 (100.0) | 14 (93.3) | 15 (93.8) | 56 (96.6) |
| **Gender** | Female | 12 (92.3) | 11 (78.6) | 13 (86.7) | 10 (62.5) | 46 (79.3) |
|  | Genderqueer | 0 (0.0) | 0 (0.0) | 0 (0.0) | 0 (0.0) | 0 (0.0) |
|  | Male | 1 (7.7) | 3 (21.4) | 2 (13.3) | 6 (37.5) | 12 (20.7) |
|  | Transgender Female | 0 (0.0) | 0 (0.0) | 0 (0.0) | 0 (0.0) | 0 (0.0) |
|  | Transgender Male | 0 (0.0) | 0 (0.0) | 0 (0.0) | 0 (0.0) | 0 (0.0) |
| **Race** | African American/Black | 0 (0.0) | 0 (0.0) | 2 (13.3) | 1 (6.3) | 3 (5.2) |
|  | American Indian/Alaska Native | 0 (0.0) | 1 (7.1) | 0 (0.0) | 0 (0.0) | 1 (1.7) |
|  | Asian | 0 (0.0) | 0 (0.0) | 0 (0.0) | 0 (0.0) | 0 (0.0) |
|  | Caucasian/White | 13 (100.0) | 13 (92.9) | 13 (86.7) | 15 (93.8) | 54 (93.1) |
|  | Native Hawaiian or Other Pacific Islander | 0 (0.0) | 0 (0.0) | 0 (0.0) | 0 (0.0) | 0 (0.0) |
|  | Other | 0 (0.0) | 0 (0.0) | 0 (0.0) | 0 (0.0) | 0 (0.0) |

| **Supplemental Table 4.** Summary of community data dashboards SUS survey responses, by research site (n=58) | | | | | | | | | | | | |
| --- | --- | --- | --- | --- | --- | --- | --- | --- | --- | --- | --- | --- |
| **State** | **Statistic** | **Question*** | | | | | | | | | | **Total Score**** |
|  |  | **PPQ04** | **PPQ05** | **PPQ06** | **PPQ07** | **PPQ08** | **PPQ09** | **PPQ10** | **PPQ11** | **PPQ12** | **PPQ13** |  |
| **KY** | MAX | 4.00 | 4.00 | 4.00 | 4.00 | 4.00 | 4.00 | 4.00 | 4.00 | 4.00 | 4.00 | 97.50 |
|  | MEAN | 2.85 | 3.00 | 3.08 | 2.92 | 3.08 | 3.00 | 3.00 | 3.23 | 3.15 | 3.00 | 75.77 |
|  | MEDIAN | 3.00 | 3.00 | 3.00 | 3.00 | 3.00 | 3.00 | 3.00 | 3.00 | 3.00 | 3.00 | 75.00 |
|  | MIN | 2.00 | 2.00 | 1.00 | 1.00 | 2.00 | 1.00 | 2.00 | 2.00 | 2.00 | 1.00 | 65.00 |
|  | N | 13.00 | 13.00 | 13.00 | 13.00 | 13.00 | 13.00 | 13.00 | 13.00 | 13.00 | 13.00 | 13.00 |
|  | STD | 0.69 | 0.58 | 0.76 | 0.95 | 0.49 | 0.71 | 0.41 | 0.60 | 0.55 | 0.82 | 10.43 |
| **MA** | MAX | 4.00 | 4.00 | 4.00 | 4.00 | 4.00 | 4.00 | 4.00 | 4.00 | 4.00 | 4.00 | 97.50 |
|  | MEAN | 3.00 | 3.00 | 3.14 | 2.79 | 3.00 | 2.79 | 2.29 | 2.64 | 2.93 | 2.50 | 70.18 |
|  | MEDIAN | 3.00 | 3.00 | 3.00 | 3.00 | 3.00 | 3.00 | 2.50 | 3.00 | 3.00 | 3.00 | 68.75 |
|  | MIN | 1.00 | 1.00 | 1.00 | 1.00 | 2.00 | 1.00 | 0.00 | 1.00 | 1.00 | 1.00 | 32.50 |
|  | N | 14.00 | 14.00 | 14.00 | 14.00 | 14.00 | 14.00 | 14.00 | 14.00 | 14.00 | 14.00 | 14.00 |
|  | STD | 0.96 | 0.88 | 0.77 | 0.97 | 0.68 | 0.70 | 1.38 | 0.84 | 1.07 | 1.16 | 16.30 |
| **NY** | MAX | 4.00 | 4.00 | 4.00 | 4.00 | 4.00 | 4.00 | 4.00 | 4.00 | 4.00 | 4.00 | 92.50 |
|  | MEAN | 2.67 | 3.40 | 3.27 | 3.33 | 3.13 | 2.80 | 2.87 | 3.13 | 3.00 | 3.27 | 77.17 |
|  | MEDIAN | 3.00 | 3.00 | 3.00 | 4.00 | 3.00 | 3.00 | 3.00 | 3.00 | 3.00 | 3.00 | 75.00 |
|  | MIN | 0.00 | 2.00 | 2.00 | 1.00 | 2.00 | 1.00 | 2.00 | 2.00 | 2.00 | 2.00 | 52.50 |
|  | N | 15.00 | 15.00 | 15.00 | 15.00 | 15.00 | 15.00 | 15.00 | 15.00 | 15.00 | 15.00 | 15.00 |
|  | STD | 1.05 | 0.63 | 0.59 | 0.90 | 0.74 | 0.77 | 0.52 | 0.74 | 0.76 | 0.80 | 11.41 |
| **OH** | MAX | 4.00 | 4.00 | 4.00 | 4.00 | 4.00 | 4.00 | 4.00 | 4.00 | 3.00 | 4.00 | 82.50 |
|  | MEAN | 2.13 | 2.81 | 2.81 | 3.06 | 2.94 | 3.00 | 2.44 | 2.69 | 2.38 | 2.69 | 67.34 |
|  | MEDIAN | 2.00 | 3.00 | 3.00 | 3.00 | 3.00 | 3.00 | 2.50 | 3.00 | 3.00 | 3.00 | 67.50 |
|  | MIN | 0.00 | 1.00 | 1.00 | 2.00 | 2.00 | 1.00 | 1.00 | 1.00 | 1.00 | 1.00 | 45.00 |
|  | N | 16.00 | 16.00 | 16.00 | 16.00 | 16.00 | 16.00 | 16.00 | 16.00 | 16.00 | 16.00 | 16.00 |
|  | STD | 1.02 | 0.75 | 1.11 | 0.57 | 0.68 | 0.82 | 0.96 | 0.87 | 0.81 | 0.79 | 12.33 |
